# Grape Skin Composting Process to Recycle Food Waste: Kinetics and Optimization

**DOI:** 10.3390/foods13060824

**Published:** 2024-03-07

**Authors:** Tea Sokač Cvetnić, Korina Krog, Katarina Lisak Jakopović, Davor Valinger, Jasenka Gajdoš Kljusurić, Maja Benković, Tamara Jurina, Tamara Jakovljević, Ivana Radojčić Redovniković, Ana Jurinjak Tušek

**Affiliations:** 1 Faculty of Food Technology and Biotechnology, University of Zagreb, Pierottijeva 6, 10 000 Zagreb, Croatia; tsokac@pbf.hr (T.S.C.); kkrog@pbf.hr (K.K.); klisak@pbf.unizg.hr (K.L.J.); davor.valinger@pbf.unizg.hr (D.V.); tamara.jurina@pbf.uniz.hr (T.J.); irredovnikovic@pbf.unizg.hr (I.R.R.); 2 Croatian Forest Research Institute, Cvjetno naselje 41, 10 450 Jastrebarsko, Croatia; tamaraj@sumins.hr

**Keywords:** food waste, grape skins, composting, degradation, optimization, compost kinetic

## Abstract

Within the various approaches to organic waste handling, composting has been recognized as an acceptable method to valorize organic waste. Composting is an aerobic technique of microbial disruption of organic matter which results with compost as a final product. To guarantee the quality of the compost, key process factors (like the moisture content, temperature, pH, and carbon-to-nitrogen ratio) must be maintained. In order to optimize the process, nine composting trials using grape skins were conducted in the present study under various initial moisture content and air flow rate conditions over the course of 30 days. The processes were monitored through physicochemical variables and microbiological activity. Also, the kinetics of the organic matter degradation and microbial growth were investigated. Although the thermophile phase was only achieved in experiments 3 and 8, the important variables proved the efficiency of all nine composting processes. The organic carbon content and C/N ratio decreased after the 30 days of composting processes and a great color change was noticed too. The values for the germination index for all experiments were above 80%, which means that the final products are non-toxic for plants. Also, the greatest change in organic carbon content in was evident in experiment 3; it decreased from 71.57 to 57.31%. And consequently, the rate of degradation for that experiment was the highest, at 0.0093 1/day. Furthermore, the response surface methodology was used to identify optimal operating conditions for grape skin composting and the obtained conditions were 58.15% for the initial moisture content and 1.0625 L/min for the air flow rate.

## 1. Introduction

The rising necessity for the supply of food to meet the needs of the world’s rapidly expanding population density is driving the need for ecologically sound and efficient methods of managing food waste [1]. Grapes (*Vitis vinifera* L.) are an important fruit crop in Mediterranean countries, and their annual production is around 29 million tons [2,3]. Around 70% of grapes produced are used for wine production. During wine production, precisely after pressing the whole grapes, grape pomace is produced in amounts of 20–25% of the total mass of the pressed grapes, and it is composed of grape skins, seeds, and stalks [4]. Furthermore, grape pomace is not only rich in organic matter, but also in polyphenols and organic acids (such as tartaric, malic, and citric), and disposing of this waste on landfills presents a serious threat to the environment [5]. When disposed on landfills, the decomposition of organic waste takes place and leads to the production of odor and leachate, which endanger both the ecosystem and people’s well-being [2]. Thus, there is an urgent economic and environmental need to develop technologies for the management of organic wastes [6].

Composting has been considered the most effective and environmentally friendly technique in which organic waste is recycled and transformed into a “compost” product abundant in nutrient content with a low prevalence of pathogenic microorganisms [6,7]. It is a dynamic and biological process in which organic substrate is degraded by numerous microorganisms including actinomycetes, bacteria, and fungi, in the presence of oxygen [7]. During the composting process, due to microbial activity, heat is generated and organic substrate passes through a thermophilic phase (temperatures above 45 °C) resulting in a stable final product free of pathogens that can be applied to land [8]. With the exception of a solid product, compost, the process also results in by-products such as carbon dioxide, ammonia, and water [9]. In order to achieve a good-quality and stable compost, the process should be performed well, including the monitoring of variables such as moisture content, pH, C/N ratio, temperature, and aeration rate, all of which are recognized as important variables during the process performance [6,10,11]. An inadequately executed composting procedure yields immature compost, or inadequately stabilized organic matter, which can negatively impact plant growth and the soil environment, serve as a disease source, and harm crops via phytotoxicity [12]. Compost’s maturity and stability are connected to its quality. While maturity refers to a product’s ability to be utilized effectively in agriculture and is related to features of phytotoxicity and plant growth, stability relates to a product’s organic matter’s resistance to extensive degradation or higher microbiological activity [13]. In order to obtain high-quality compost, it is necessary to optimize composting process conditions [14]. There are examples of single-factor optimization of municipal solid waste composting processes [15], such as palm oil mill effluent composting [16], meddler pruning waste composting [17], biodegradable solid waste composting [18], and food waste composting [14]. The one-factor-at-a-time method is easy to use but it has several disadvantages. The main drawback of this approach is that it ignores the interactions between the variables under investigation. Consequently, the full effects of the variables on the response are not depicted by this technique. The need for more experiments to complete the research results in higher time and expense requirements as well as higher material and reagent consumption, which is another drawback of one-factor optimization [19,20]. The efficient alternative to the one-factor-at-a-time method is the use of multivariate statistical and mathematical tools like Response Surface Methods (RSMs) coupled with design of experiments (DOEs). By examining the response of several variables at once, it makes it possible to optimize the experimental setup. In addition, the RSM provides an algebraic framework that forecasts system dynamics, facilitating effective allocation of resources and making choices. The RSM can also be used to determine the ideal experimental mixture [21]. The RSM has certain drawbacks, though, when applied to scientific investigation. One drawback of the RSM is that it relies on the assumption of a linear connection among variables, which complicated systems may not always exhibit. Moreover, obtaining the massive quantity of data that the RSM needs for properly modeling the system may be expensive and take time [22]. Notwithstanding these shortcomings, the RSM’s capacity to forecast system dynamics and optimize experimental settings makes it a useful method in studies. Response Surface Methods coupled with design of experiments have been also applied in the optimization of composting procedures. For example Iqbal et al. [23] coupled Box-Behnken DOE with RSM for optimisation of kitchen waste composting, Sayara et al. [24] and Cabeza et al. [25] applied central composite design and RSM for optimisation of municipal solid waste composting, Mohd Sokri et al. [26] used central composite design (CCD) and RSM for optimization of co-composting of horse manure with pineapple waste, Younesi et al. [27] applied CCD with RSM to improve the efficiency of compost leachate treatment, while Sharma et al. [28] coupled CCD and RSM to maximize the amounts of cattle faeces and waste from flowers combined when composting in an agitated pile. Furthermore, Kazemi et al. [29] applied two-level factorial design to study the effect of four variables on the maturity, stability and toxicity of the municipal solid waste compost.

There is a large variety of currently available compost systems, and they fall into two primary types: open systems, such as windrows and piles, and closed systems, such as reactors and composters [30]. Open systems require manually or mechanically turning the pile to provide sufficient oxygen supply for microbial degradation of organic matter. The main advantages of this system are low cost and simple operation, but on the other hand, it is difficult to control the process variables which makes the composting time longer. The closed systems have advantages over the open ones: they take less space and provide better control of the process, which leads to high efficiency of the process [30,31].

As mentioned before, organic wastes are broken down by mesophilic and thermophilic bacteria, while fungi contribute to the composting process by producing distinct functional enzymes and breaking down different kinds of molecules [32]. To define the optimal conditions for microbial activity, the relationship between the composting rate and environmental conditions should be analyzed and defined through kinetic studies. As described by Hamelers [33], composting kinetics is the analysis of the interrelation of the composting rate on external influences over a broad range of practically significant variables using a comprehensive system of equations. Inductive kinetic models are based on the breakdown of organic matter because it provides the free energy required to drive the process and should be able to predict the processing rate [34]. Substrate degradation models, in which the amount or concentration of residual substrate serves as the independent variable, are among the most widely used techniques for simulating the kinetics of composting [35]. It is possible to model the decomposition rate using a zero-order [36,37], first-order [35,38], second-order [36,39,40] and n-order [36,40] differential equation.

Based on the above mentioned in this study, nine composting experiments of grape skins were completed in laboratory reactors under particular settings of initial moisture content of substrate (50–65%) and air flow rate (0.350–1.700 L/min). Moisture content has an impact on microbial activity and, finally, on the rate of degradation. For microbial activity, it is necessary to ensure the appropriate oxygen content for the effective degradation of organic matter. During 30 days, the processes were monitored through physicochemical and microbiological characteristics. To our best knowledge, this is the first study dealing with the optimization of the in-vessel grape skin composting process. Furthermore, this is the first study analyzing the kinetics of organic matter degradation during grape skin composting.

## 2. Materials and Methods

### 2.1. Materials

#### 2.1.1. Grape Skin

The skin of the white grape pomace *Vitis vinifera* cv. Graševina, harvested in 2021 (Kutjevo, Croatia), was used as a raw material for the composting process. Grape pomace was stored in a freezer at −18 °C. Before the performance of the experiments, grape skins were separated from seeds and stalks by sieving. Due to pH value in acidic range of the raw grape skin which is unacceptable for composting, the 10% sodium hydrogen carbonate solution was added to adjust the pH [41]. After adding the solution, skins were left at room temperature during the night.

#### 2.1.2. Chemicals

The sodium hydrogen carbonate was purchased from Kemika (Zagreb, Croatia). Sodium chloride was from Sigma Aldrich (St. Louis, MO, USA). The incubation media for isolation of fungi was Sabouraud dextrose agar purchased from Liofilchem (Roseto degli Abruzzi, Italy) and for isolation of bacteria was Tryptic glucose yeast agar purchased from Biolife Italiana (Monza, Italy).

### 2.2. Methods

#### 2.2.1. Composting Process

The composting processes were performed based on the settings specified by the full-factorial experimental design (Table 1). The effect of the moisture content of the substrate (50–65%) and air flow rate (0.35–2.0 L/min) on how well the composting process works was investigated. The composting processes of grape skin (m = 1.9 kg) were performed in laboratory batch reactors in a total volume of V = 5 L. The dimensions of the reactors were as follows: diameter, d = 16 cm, and height, h = 25 cm. The reactors were isolated with a wall thickness of 5 cm. Over the course of the thirty days of the composting process, the reactors were aerated with a constant air flow rate to ensure aerobic conditions. The thermometers were placed in the center of the substrate to monitor the temperatures constantly. After every 48 h, the samples were taken for physicochemical analysis and after every 72 h for microbiological analysis from the middle point of the composting mass.

#### 2.2.2. Physicochemical Analysis of the Compost Samples

##### Moisture and Dry Matter Content of Compost Samples

The moisture and dry matter content were determined by drying the samples for 24 h at 105 °C in a dryer (Inkolab ST60T, Zagreb, Croatia) [42]. A certain mass of the sample (m = 2 ± 0.001 g) was weighed into metal containers, and after drying the containers were placed in a desiccator where they were cooled to room temperature. The difference between the mass before and after drying is the proportion of moisture content. Three repetitions of the measurements were made, and the outcomes are shown as the mean value ± standard deviation.

##### Total Organic Matter and Ash Content of Compost Samples

Total organic matter and ash content were determined by heating the samples after drying at 550 °C for 5 h in a muffle oven (B410, Nabertherm, Lilienthal, Germany). The percentage of loss of volatile substances was expressed as a share of total organic matter, while the mass remaining after burning was expressed as the ash fraction [43]. Three repetitions of the measurements were made, and the outcomes are shown as the mean value ± standard deviation.

##### Carbon and Nitrogen Content of Compost Samples

Total carbon and nitrogen content were determined by an elemental analyzer with a spectrophotometer (LaboMed UV-VIS, Los Angeles, CA, USA) according to the method described by Lovreškov et al. [44]. Measurements were performed with three repetitions, and the results are presented as the mean value ± standard deviation.

##### pH, Conductivity and Total Dissolved Solids of Compost Samples

Compost and distilled water were combined in a ratio of 1:10 (*w*/*v*) to create the extracts, which were then stirred at 150 revolutions per minute for an hour using a magnetic stirrer. Following extraction, the resulting mixture was filtered [43]. In the filtrate, the pH value was determined using a pH meter (914, Metrohm, Herisau, Switzerland) and the conductivity and total dissolved solids using a conductometer (SevenCompact, MettlerToledo, Greifensee, Switzerland). Three repetitions of the measurements were made, and the outcomes are shown as the mean value ± standard deviation.

##### Color Change of the Compost Samples and Compost Extracts

The color of all composts and compost extracts was determined using a PCE-CSM3 colorimeter (PCE Instruments, Meschede, Germany). According to Hunter’s color coordinates, L* represents light, a* represents the range from green to red, and b* represents the range from blue to yellow. The values a* and b* are used for the calculation of the Hue angle and Chroma value [45]. The total color change of the compost and corresponding compost extracts (Δ*E*) was determined according to Equation (1):

(1) $$\Delta E=\sqrt{{\left(L^{*}-L_{0}^{*}\right)}^{2}+{\left(a^{*}-a_{0}^{*}\right)}^{2}+{\left(b^{*}-b_{0}^{*}\right)}^{2}}$$

where *L*_0_, *a*_0_, and *b*_0_ are the values of the Hunter coordinates of the samples/extracts of the initial substrate samples, and L*, a*, and b* are the values of the Hunter coordinates of the compost/compost extracts during the composting process. Three repetitions of the measurements were made, and the outcomes are shown as the mean value ± standard deviation.

#### 2.2.3. Microbiological Analysis of the Composting Process

Firstly, the isolation media for microorganisms was prepared according to the instructions on the packaging. The isolation media for fungi were prepared by dissolving the 65 g of Sabouraud Dextrose Agar in 1 L of distilled water and heated until the powder is completely dissolved. The isolation media for bacteria were prepared by dissolving the 23 g of Tryptic glucose yeast agar in 1 L of distilled water and heated until the powder is completely dissolved. Both media were sterilized by autoclaving at 121 °C for 15 min. Before using for the viable count, the media were cooled to 47–50 °C.

The viable count of the bacteria and fungi during the composting process was determined as described by Sokač et al. [46], with some modifications. Microorganisms were monitored every 72 h. An amount of 5 g of milled compost sample was added to 100 mL of sterile saline solution, and the suspension was mixed on a shaker at 100 rpm (685/2, Lab Medical, Loos, France) for 1 h. After the extraction time, the suspension was filtered through 100% cellulose filter paper (pore size 5–13 µm, LLG Labware, Meckenheim, Germany) to separate the aqueous extract from the solid phase. The filtrate was used to prepare the appropriate decimal dilution. The viable plate count was determined by inoculating 1 mL of dilution on a medium for growth of bacteria or fungi. The Petri dishes were incubated in a thermostat (561-08/2, InkoLab, Zagreb, Croatia) at 28 °C for fungi and at 37 °C for bacteria for 5 days. The results were expressed as CFU/g of dry matter.

#### 2.2.4. Study of the Germination Index (*GI*)

The germination test was performed every five days with 20 salad seeds as described by Hashemi et al. [47]. Subsequently, 5 mL of compost extracts were added to filter papers in Petri dishes, and one set of filter papers was made with distilled water as a control. Each set contained twenty salad seeds, which were then incubated for five days at 25 °C. The number of germinating seeds and the root elongation of the samples were measured. Finally, the *GI* was calculated using Equation (2):

(2) $$GI=\frac{G_{S}\cdot L_{S}}{G_{C}\cdot L_{C}}\cdot 100$$

where *G*_S_ is the seed germination (%) and *L*_S_ is the root elongation (mm) for the compost sample, and *G*_C_ and *L*_C_ correspond to control values [47]. Measurements were performed with three repetitions, and the results are presented as the mean value ± standard deviation.

#### 2.2.5. Bulk Density and Porosity of the Compost

The bulk density of a final compost sample was determined according to a method described by Buljat et al. [48]. The volumeter works on the principle of compressing the material by vibrations that squeeze the air between the particles, and as a consequence, the volume decreases and bulk density increases. The final compost sample was poured into a graduated plastic container of predetermined weight, and the mass and volume of the compost sample were recorded. The analysis was done in triplicate, and the results are expressed as the mean value ± standard deviation.

Using the known density of water (*ρ*_w_ = 1000 kg/m^3^) and estimated densities of organic matter (*ρ*_OM_ = 1600 kg/m^3^) and ash (*ρ*_ash_ = 2500 kg/m^3^), compost porosity (*ɛ*) was calculated. If the moisture content (MC), dry matter (DM), organic matter (OM), and wet bulk density (*ρ*_wb_) of the samples are known, the porosity can be calculated using the following equation [49]:

(3) $$ɛ\left(\%\right)=1-\rho_{wb}\left[\frac{MC}{\rho_{w}}+\frac{DM\cdot OM}{\rho_{OM}}+\frac{DM\cdot \left(1-OM\right)}{\rho_{ash}}\right]\cdot 100$$

#### 2.2.6. Statistical Analysis

Basic statistical analysis was performed using Statistica 14.0 (Tibco Software Inc, Palo Alto, Santa Clara, CA, USA). The differences between the means of the physicochemical characteristics during the composting process of the grape skin were tested using analysis of variance (ANOVA) at the significance level of *p* < 0.05, followed by Tukey’s HSD test.

#### 2.2.7. Organic Matter Degradation Kinetics

The degradation of organic matter was expressed as a function of time following the first order kinetic (Equation (4)) [50]:

(4) $$\frac{d(OM)}{dt}=-k\cdot OM$$

where *OM* is an amount of biodegradable solids (%) at time *t* (day) of composting process, and *k* is degradation rate (1/day).

Kinetic parameters were estimated by fitting the experimental data directly to the differential equation using the Parametric NDSolve algorithm implemented in WR Mathematica 10.0. The goodness of fit of the developed models was assessed using the Root Mean Square Value (RMSE) (Equation (5)), the Reduced Chi-square Value (c^2^) (Equation (6)), and modeling efficiency (EF) (Equation (7)):

(5) $$RMSE=\sqrt{\sum_{i=1}^{n}\frac{{\left({OM}_{pred,i}-{OM}_{exp,i}\right)}^{2}}{N}}$$

(6) $$\chi^{2}=\frac{\sum_{i=1}^{N}{\left({OM}_{exp,i}-{OM}_{pred,i}\right)}^{2}}{N-n}$$

(7) $$EF=\frac{\sum_{i=1}^{n}{\left({OM}_{exp,i}-{OM}_{exp,mean}\right)}^{2}-\sum_{i=1}^{n}{\left({OM}_{pred,i}-{OM}_{exp,i}\right)}^{2}}{\sum_{i=1}^{n}{\left({OM}_{exp,i}-{OM}_{exp,mean}\right)}^{2}}$$

where *OM*e_xp_ is the experimental organic matter amount, *OM*_exp,mean_ is the mean value of the experimental organic matter amount, the *OM*pred kinetic model predicts the organic matter amount, N is the number of experimental data points, and n is the number of model parameters.

#### 2.2.8. Optimization of Composting Process Conditions Using Response Surface Method

The relationship between initial moisture content (*X*_1_), air flow rate (*X*_2_), and compost organic matter amount (*Y*) after 30 days of composting was analyzed. The effect of all variables was analyzed according to the experimental design (Table 1). A total of 9 experiments were conducted randomly. Second-order polynomial equations were used to fit the experimental data (Equation (8)):

(8) $$Y=\beta_{0}+\beta_{1}\cdot X_{1}+\beta_{2}\cdot X_{2}+\beta_{11}\cdot X_{1}^{2}+\beta_{22}\cdot X_{2}^{2}+\beta_{12}\cdot X_{1}\cdot X_{2}$$

where *Y* is the predicted response, *β*_0_ is the constant, *β*_1_ and *β*_2_ are the linear coefficients, *β*_11_ and *β*_22_ are the quadratic coefficients, and *β*_12_ are the cross-product coefficients. RSM was carried out by employing Statistica 14.0 software package. (TIBCO^®^ Statistica, Palo Alto, Santa Clara, CA, USA). The suggested RSM models were used to predict the most suitable composting settings more accurately.

## 3. Results and Discussion

In the present study, nine different composting experiments on grape skin were carried out in laboratory reactors over the course of 30 days. Besides temperature, the processes were monitored through important physicochemical variables (such as moisture and dry matter content, organic matter content, ash content, carbon and nitrogen content, C/N ratio, pH, conductivity, total dissolved solids, color change of compost samples and their extracts) and microbiological characteristics. Also, the germination test of the compost was carried out. At the end of the process, the bulk density and porosity of the final product were determined.

### 3.1. Composting Temperature

Temperature is considered a critical variable for the composting process, and it passes over four temperature phases where distinct microorganism populations are predominant in each phase. Among these periods are the mesophilic, thermophilic, cooling, and maturation phases [6,7]. In the mesophilic phase, energy-dense and readily broken-down substances like sugars and proteins are degraded by mesophilic fungi, bacteria, and actinomycetes. As a result of microbial activity, heat is generated and the temperature increases, passing from the mesophilic phase (25–45 °C) to the thermophilic phase (45–65 °C) [6]. Higher temperatures are desirable because they ensure waste sanitation, rapid degradation, and humification. But on the other hand, temperatures in this phase should not exceed 70 °C because they slow microbial activity and can cause enzyme denaturation [51]. In this phase, dominant bacteria and fungi are adapted to higher temperatures [52]. Furthermore, with time, the high-dense substances become spent, the temperature declines, and mesophilic microorganisms dominate repeatedly. The maturation phase takes place at lower temperatures, but reactions are still occurring despite the low microbial activity [6]. In this phase, non-biodegradable substances, like lignin-humus complexes, take center stage [52].

**Figure 1:**
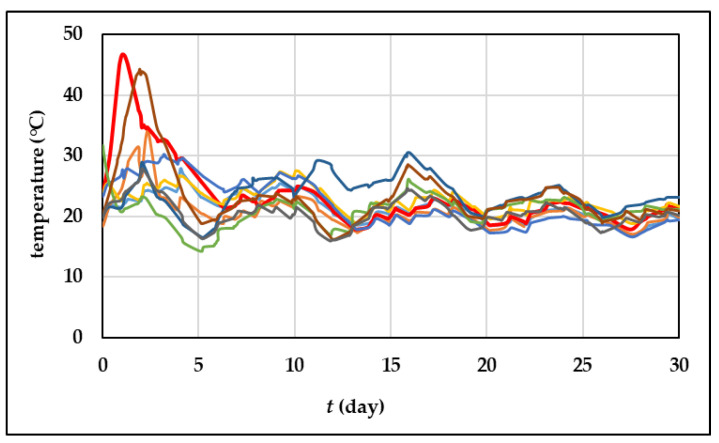
Temperature changes during the 30 days of grape skins composting processes (**—** experiment 1; **—** experiment 2; **—** experiment 3; **—** experiment 4; **—** experiment 5; **—** experiment 6; **—** experiment 7; **—** experiment 8; **—** experiment 9).

Temperature profiles for the composting processes are shown in Figure 1. All experiments showed a quick rise in temperature, and the lack of a lag phase indicates that the substrate and the common aerobic microorganisms have a good and ready affinity, as previously described by Perra et al. [53]. The thermophilic phase was achieved in the first three days of the process. According to Oviedo-Ocana et al. [54], the quick rise in temperature indicates that the settings for the technique involving the substrate under study are suitable (i.e., pH, moisture, and porosity). As shown, only in two composting processes (experiment 3 where the initial moisture content was 65% and the aeration rate was 0.88 L/min and experiment 8 where the initial moisture content was 57.5% and the aeration rate was 0.43 L/min) was the thermophile phase achieved, the temperature was above 45 °C, and it lasted for approximately 30 h. The presented results show that for the experiment with an initial moisture content of 50%, the composting temperature did not exceed 31 °C. The findings gathered are consistent with the description by Jain et al. [55] where it was described that moisture content is a crucial variable that affects how waste materials’ physical, chemical, and biological characteristics change. Furthermore, according to Zahrim et al. [56], the lack of green waste and the small size of the reactor could also result in composting temperatures under 40 °C. But according to Paradelo et al. [57], the thermophilic phase at temperatures under 45 °C does not mean that there was no degradation, because the visual change in the fresh grape skins and composted material was noticed, and also, the other variables can prove the degradation, such as organic matter content and the C/N ratio.

### 3.2. Physicochemical Analysis of the Compost Samples

The results of the physicochemical analysis of the compost samples and their extracts during the composting processes are shown in the sections below. Differences in physicochemical variables between reactors and the composting days are statistically analyzed and the results are given in Supplementary Table S1.

#### 3.2.1. Moisture Content and Dry Matter Content

**Figure 2:**
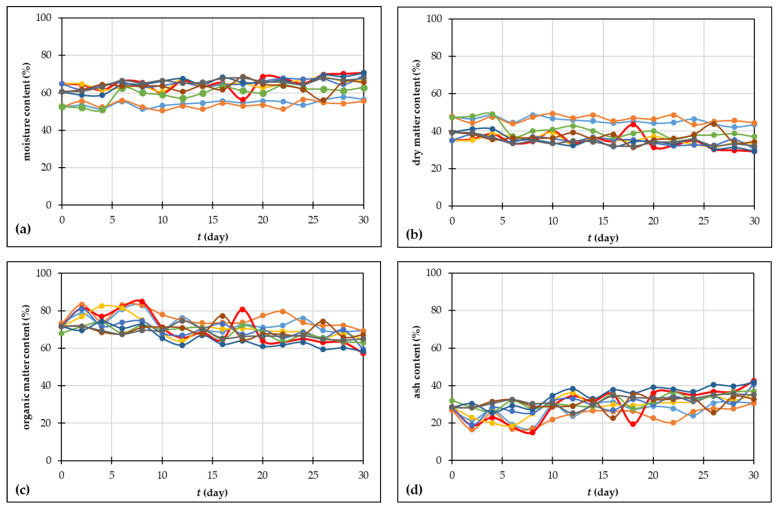
Changes in (**a**) moisture content; (**b**) dry matter content; (**c**) organic matter content; (**d**) ash content during the 30 days of grape skin composting process (• experiment 1; • experiment 2; • experiment 3; • experiment 4; • experiment 5; • experiment 6; • experiment 7; • experiment 8; • experiment 9).

Moisture content influences microbial activity, oxygen uptake rate, free air space, temperature of the process, and the rate of organic matter degradation [58,59]. Additionally, water is used by microbes to move nutrients and energy components across their cell membranes [51]. At the beginning of the processes, the moisture content was between 50–62% which is in accordance with the optimal moisture content for the composting process [30,58]. Furthermore, the appropriate level of moisture fluctuates and is contingent upon the composting material due to the unique physical, chemical, and biological characteristics of the materials [51]. A lower moisture content, less than 30%, might result in the substrate losing moisture throughout composting, and that can hinder biological activity. Conversely, though, the moisture content should not exceed 65–70%, which might restrict oxygen flow, since the spaces between pores are closed and the process can become anaerobic [51,59]. But as described by Abdallah et al. [60], the wide range of published values indicates that there is no optimal moisture content for composting materials that is generally applicable. The same authors [60] mentioned that the connection among moisture level and water availability, particle size, porosity, and permeability is impacted by the unique physical, chemical, and biological properties of every substance. As shown on Figure 2a, during all composting experiments, moisture content increased slightly, and after 30 days, it was between 55–70%. At the end of the composting process, the highest moisture content was achieved for experiment 3 (70.776 ± 0.028%), followed by experiment 8 (70.630 ± 2.874%) and experiment 5 (69.332 ± 0.096%). It is also important to mention that according to the results obtained in this study, moisture content change is quite slow, and the differences regarding initial moisture content values were notable after approximately 20 days of composting (see Supplementary Materials). As mentioned before, moisture content change is linked to temperature change during the composting process. Microorganisms degrade organic matter when there is oxygen, and metabolic water is released [51] which agrees with the results obtained in this study. The dry matter content in the beginning was 35–47%, and at the end of the process, it was 29–43% (Figure 2b). Considering the statistical analysis shown in the Supplementary Section, it can be noticed that significant differences between initial and final moisture content and initial and final dry matter content were noticed in the above-mentioned experiments 3 and 8.

#### 3.2.2. Organic Matter Content and Ash Content

Organic matter degradation and ash content in grape skin were monitored during composting processes (Figure 2c,d). The initial organic matter content was in a range from 67.964 ± 1.084% (experiment 7) to 73.179 ± 0.576% (experiment 1), and at the end of the processes, it was in a range from 57.319 ± 0.129% (experiment 3) to 69.273 ± 0.824% (experiment 2). The obtained results show that during experiment 3, which was carried out under an initial moisture content of 65% and an air flow rate of 0.88 L/min, the greatest reduction in organic matter was achieved. For the described experiment 3, statistical analysis showed a significant difference in organic matter content after 12 days of composting, and that difference increased during the composting period (Supplementary Materials). During composting, various biochemical reactions occur, transforming the complex compounds into simpler components, and the consequence is a reduction in organic matter content [43]. Ash, part of the material that is inorganic, includes inorganic minerals like magnesium, iron, calcium, and sodium, along with other trace metals. In general, organic matter content and ash content are reciprocal, with high organic matter content resulting in lower ash content [43]. The results obtained in this work are in agreement with the literature [61]. The initial grape skin had a higher content of organic matter and lower values of ash content. After 30 days of composting processes, organic matter decreased and the ash content increased. As for the organic matter content, statistical analysis showed a significant difference in ash content after 12 days of composting, and that difference increased during the composting period (Supplementary Materials).

#### 3.2.3. Total Carbon and Nitrogen Content, C/N Ratio

**Figure 3:**
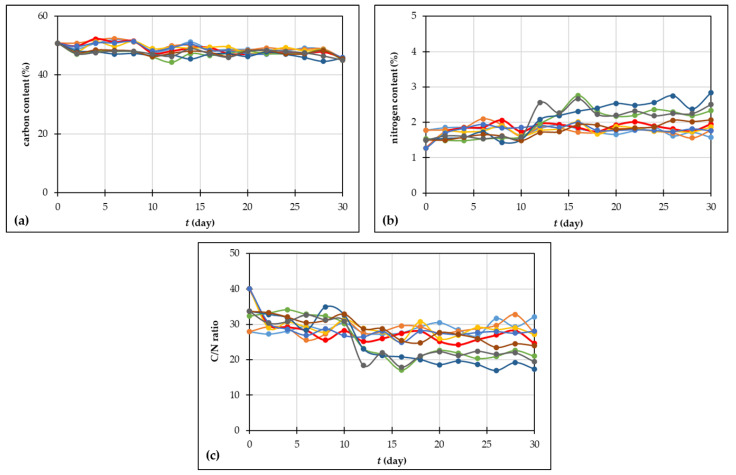
Changes in (**a**) carbon content; (**b**) nitrogen content; (**c**) C/N ratio during the 30 days of grape skin composting process (• experiment 1; • experiment 2; • experiment 3; • experiment 4; • experiment 5; • experiment 6; • experiment 7; • experiment 8; • experiment 9).

Carbon is one of the primary components of organic waste, and in composting processes, it decreases because of the deterioration of organic matter and is lost as carbon dioxide [58]. Otherwise, the nitrogen content increases due to mineralization and the production of ammonium and nitrate [51]. Carbon serves as a source of energy, and nitrogen is needed to construct cell structures [31,58]. Azim et al. [51] reported that the total nitrogen before composting is 1–4% of the total dry weight of compost. In this study, the initial value of total carbon was 50.8% (Figure 3a) and the initial values of nitrogen content were between 1.27% (experiment 3) and 1.78% (experiment 2) (Figure 3b).

The C/N ratio is a necessary variable for microbiological existence, and it serves as a measurement for the level of decomposition of organic matter. The elements need to be in a certain proportion to maintain ordinary microbial turnover in order to produce a product of outstanding standards [31]. According to the literature [10,31,62], the optimal C/N ratio for composting is between 25 and 30:1 for all types of organic waste, but the ratio between 20 and 40:1 is also acceptable [63]. For microorganisms to develop quickly and to guarantee adequate energy consumption, the ratio should be approximately thirty [51]. The initial C/N ratio of grape skin used in this study was in the range from 27.94 (experiment 1 and 2) to 40.07 (experiments 3–5), and similar values of C/N ratio for grape pomace were reported by Paradelo et al. [57] and Barros et al. [64]. In all experiments performed, the C/N ratio decreased. By the finish of the composting processes, the C/N values were between 17.36 (experiment 7) and 27.174 (experiment 4) (Figure 3c), which is another proof that the composting process was successful, although the thermophile phase was not achieved in most experiments. The C/N ratio change could be explained by the aeration rate. Similarly, this was noticed by Alkoaik [65] where during composting of agricultural residues (mixture of tomato plant residues and 20%-chicken manure), the *C/N* ratio was downsized from 30/1 to 23/1 in the rotating bioreactor, while it remained at 30/1 in the static bioreactor, implying that the aeration is an important factor affecting the composting process. Furthermore, in this study, the greatest change in the C/N ratio was noticed in experiment 3, in which the C/N ratio decreased from 40.07 to 24.67. In the case of experiment 3, a significant difference in carbon content and C/N ratio regarding the initial value was noticed after eight days of composting (Supplementary Materials).

#### 3.2.4. Total Color Change of Compost Samples and Corresponding Extracts

**Figure 4:**
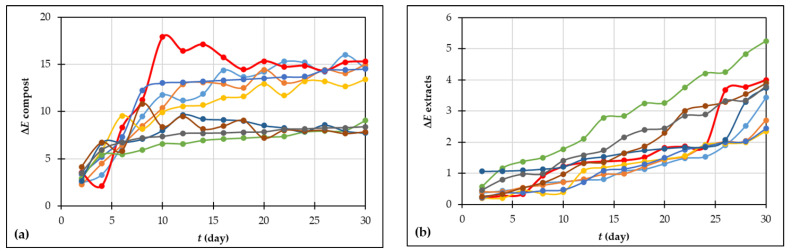
Color changes of (**a**) compost sample; (**b**) compost extracts during the 30 days of grape skin composting process (• experiment 1; • experiment 2; • experiment 3; • experiment 4; • experiment 5; • experiment 6; • experiment 7; • experiment 8; • experiment 9).

The total color change of compost samples and corresponding extracts is an evident proof of the performance of the composting procedure and changes in compost color highlight the degree of compost stabilization [65]. In general, the substrates during composting gradually turn black due to the degradation of organic matter and evolution of humic substances [66]. Figure 4 shows the total color change of compost samples and corresponding extracts during the 30 days of the composting procedure. The total color change of compost samples in a range from 2.436 to 5.910 was observed already on the second day of composting. The total color change increased until the end of the process. At the end of the composting procedure, the highest total color change of the compost was observed in experiment 3 (ΔE = 15.30), and the lowest value of total color change was measured in experiment 7 (ΔE = 7.72). Zhrim et al. [56] reported a total color change at the end of the tomato residues composting around 15.2, which agrees with the results presented in this study. Statistical analyses showed that there were significant statistical differences between samples from experiments 1, 3, 4, and 5 and samples from experiments 2, 6, 7, 8, and 9 at the end of the composting procedure. The total color change of the compost extracts followed the same trend as the compost samples. In the case of compost extracts, the total color change of the samples could be explained by the presence of dissolved and particulate organic matter [56].

#### 3.2.5. pH, Total Dissolved Solids and Conductivity

The pH value has an impact on microbiological activity as well as is an essential factor in the procedure for making compost. Nevertheless, fungi prosper in acidic surroundings, and bacteria favor a pH that is almost neutral [62]. The optimal pH range for composting is considered to be 5.5–8 [51,58]. As described by Azim et al. [51], during composting, the pH changes through four phases: (i) acid-genesis phase in which pH decreases and microorganisms produce carbon dioxide and organic acids; (ii) alkalization phase characterized by increasing pH, bacterial degradation of protein and ammonia production; (iii) pH stabilization phase in which C/N ration decreases and reactions become slower; (iv) stable phase where the pH is close to neutral and the compost is in maturation.

**Figure 5:**
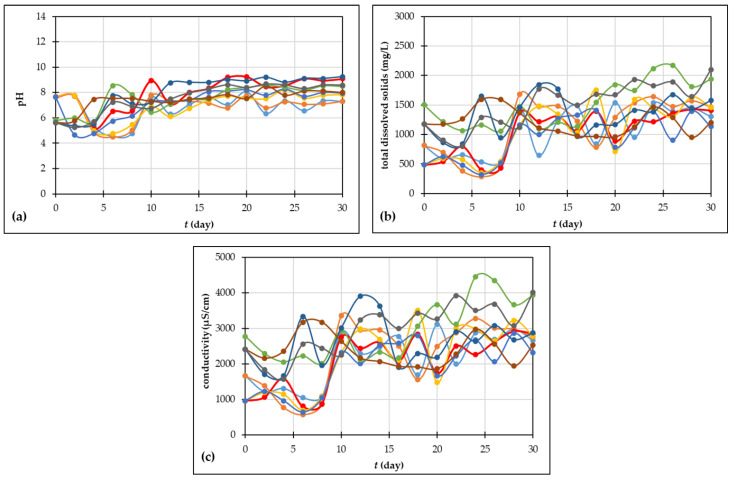
(**a**) pH; (**b**) total dissolved solids and (**c**) conductivity during the 30 days of grape skin composting process (• experiment 1; • experiment 2; • experiment 3; • experiment 4; • experiment 5; • experiment 6; • experiment 7; • experiment 8; • experiment 9).

Fresh grape skin has a pH in the acidic area, precisely 4, which makes this material unsuitable for the composting process, considering the optimal range. After adding 10% sodium hydrogen carbonate solution to the grape skin, the initial pH value was in a range of 5.62–7.68, which is acceptable for composting. The changes in pH during the procedure of making compost are shown in Figure 5a. In general, during the first days of composting, pH decreases as a result of the activity of acid-forming bacteria which degrade organic material and form organic acids as intermediate products [62]. Furthermore, the depletion of natural substances that break down quickly and mineralization led to an increase in pH [51]. After 30 days of composting processes, the pH was 7.29–9.08 and it was in the alkaline area. According to the literature [62,67], a mature compost has a pH around 6–8.5, but it depends on the composted material. Also, it is important to determine the final pH of compost, after applying it on a soil [67].

As can be noticed from Figure 5b,c, total dissolved solids and conductivity are two related variables; the greater the concentration of total dissolved solids in compost, the higher the values of conductivity [67]. For all experiments, an increase can be noticed in both TDS and S during the composting time. According to Hemidat et al. [67], the values of the conductivity of the compost are from 1–10 mS/cm which corresponds to the results obtained in this study. During the early stages of the composting process, because of the high activity of microbes and the discharge of mineral salt ions from the breakdown of organic matter, such as phosphate, conductivity and total dissolved solids are raised. In the later stages, temperature drops, mineral salts are deposited, and microorganisms and ions form stable humus and the conductivity decreases [68]. The statistical analysis showed significant differences in pH values between reactors and days of the composting processes. Also, there is a significant difference in TDS and conductivity at the beginning of the processes between reactors, and at the end of the processes, a significant difference is shown in experiments 6 and 9.

### 3.3. Microbiology of the Composting Process

Their ability to degrade the compost blend is reflected in the dynamics or succession of microbial populations during making compost. As making compost progresses, the most prevalent microbes are bacteria and fungi. These microbes aid in the breakdown of organic matter by generating a variety of hydrolytic enzymes that can break down complex molecules into water-soluble compounds. In addition, they yield easily used compounds that, when mixed with soil, improve agricultural potential and maintain the environment [69]. As mentioned before, the pH is a parameter which affects microbial activity. Bacteria prefer a nearly neutral pH and fungi develop better in an acidic environment [62].

**Figure 6:**
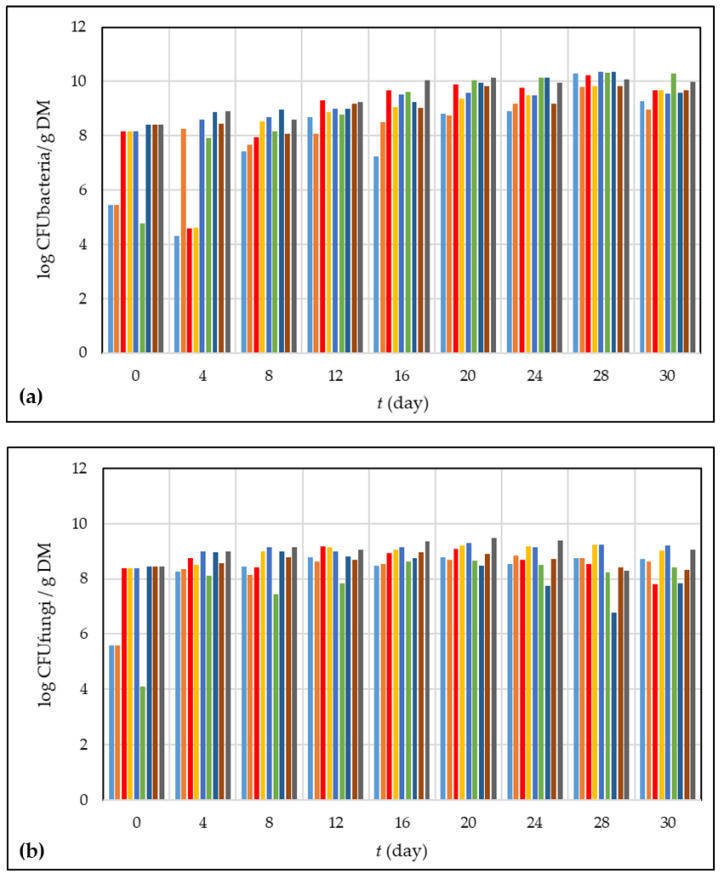
Microbial growth during the 30 days of grape skin composting process, (**a**) bacterial growth; (**b**) fungal growth (• experiment 1; • experiment 2; • experiment 3; • experiment 4; • experiment 5; • experiment 6; • experiment 7; • experiment 8; • experiment 9).

As shown in Figure 6, during the first stages of the composting process, when the pH is in the acidic range, the number of fungi is higher than the number of bacteria. Otherwise, after 8 days of the composting process, the pH is in the neutral range and lightly alkali, and the number of bacteria increases. The same trend was noticed for all nine composting experiments.

### 3.4. Germination Index (GI)

**Figure 7:**
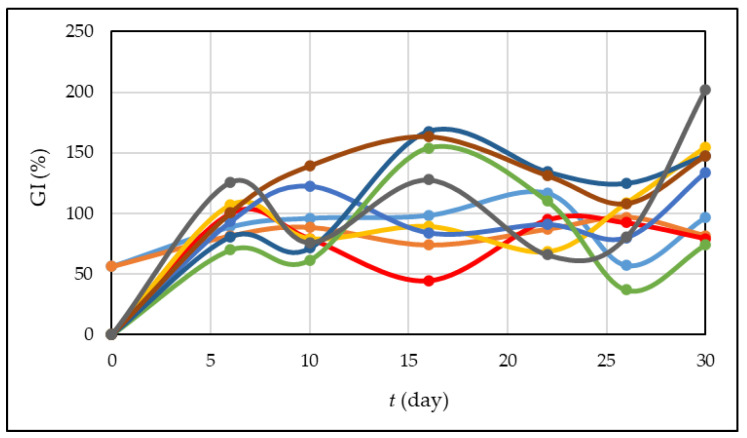
Germination index during the 30 days of the composting process (• experiment 1; • experiment 2; • experiment 3; • experiment 4; • experiment 5; • experiment 6; • experiment 7; • experiment 8; • experiment 9).

The germination index is a highly trustworthy metric for assessing the stage of maturity of organic fertilizer; it can reveal if the compost is safe, harmless, non-toxic, or useful [16]. The authors have reported that the value of GI above 80% indicates compost maturity and non-toxicity for plants [47,70]. Figure 7 presents the variations in the GI during the 30 days of composting processes. At the beginning of the processes, the GI was in the range of 0–56% which is in accordance with Perra et al. [3]. These authors carried out composting processes with different pretreated grape pomace, and they investigated the germination index of the obtained compost samples. As can be seen from Figure 7, the GI during the composting process varies, and it is due to the presence of different compounds in different stages of degradation. Studies [71,72] have shown that high salt concentrations and high organic matter, which includes organic acids, humic acid, reducing sugars, amino acids, and phenolic acids, affect the germination index. The statistical analysis showed significant differences in the germination index between experiments and days of the composting process.

### 3.5. Bulk Density and Porosity of Compost

The mass of material in a given volume is known as the bulk density of the compost, and it affects the mechanical qualities of the material, including strength, porosity, and compaction ease [73]. According to Azim et al. [51], the bulk density values for compost are often in the range of 100 to 900 kg/m^3^. Higher values imply an increase in mass and a decrease in porosity; otherwise, lower values can indicate excessive substrate aeration [51]. On the other hand, Abad et al. [74] stated that the optimal value for the compost bulk density should be <400 kg/m^3^ to be appropriate for utilizing as a growing medium. The results for the compost bulk density are shown in Table 2. The values range from 323.466 to 428.804 kg/m^3^, which is in accordance with the literature data. The highest value for the bulk density was recorded in experiment 9, which is 428.804 kg/m^3^, and the lowest values are in experiment 1, with a value of 323.466 kg/m^3^. Comparing the experimental results with the optimal range suggested by Abad et al. [74], the grape skin composts are suitable for use as a growing media for plant production. Furthermore, the porosity (pore space) depends on the bulk density and moisture content of the samples. The higher values of bulk density resulted in lower values of porosity [49]. The porosity of compost samples ranged from 61.257 to 73.563%, and similar results were obtained by Khater [49]. Also, Abad et al. [74] determined that the acceptable porosity of compost substrate should be >85%. A significant difference can be noticed in bulk density values between reactors, but there is no significant difference in porosity values.

### 3.6. Kinetics of Organic Matter Degradation

Mathematical modeling offers excellent possibilities for process analysis and optimization in order to create a method that could result in improved breakdown of organic matter and minimize the harmful effects of generated waste on the ecosystem [75]. Knowledge of the dynamic interactions among the mechanisms and laying the groundwork for a logical design process are provided by mathematical modeling. The amount of substrate (organic matter) is the main factor influencing the reaction rate in the first-order kinetics model [76]. Organic matter degradation in this work was described with a first-order kinetic model, because experimental data for organic matter change during the time following the exponential decay. However, the first-order kinetic model can be used as a useful measure for the loss of organic matter during the composting process. In Table 3, the kinetic parameters and the statistical analysis for organic matter degradation are described. As shown, the highest rate of degradation (0.0093 ± 0.0023 1/day) was estimated for experiment 3, followed by experiment 7 and experiment 4. In experiment 3, consequently, the percentage of degraded organic matter was the highest. This result can be related to the organic matter content (Figure 2c) for the mentioned experiment, where a significant decrease in organic matter was noticed after 30 days of the composting process. In all experiments, the percentage of degraded organic matter was above 70%, which confirms the performance of the composting processes. Due to the significant variability of the composition of the composting materials, it is quite difficult to compare the obtained results with the available literature. For example, Abu Qdais and Al-Widyan [35] presented organic matter degradation rates in the range of 0.0015 to 0.0055% per day in the process of agro-industrial waste, olive milling waste, grain dust, and coffee processing waste mixture composting. Furthermore, Ebrahimzadeh et al. [36] presented organic matter degradation rates in the range of 0.01 to 0.02 1/day in the process of kitchen waste, pruned elm tree branches, and sheep manure mixture composting, while Rossetti et al. [77] presented an organic matter degradation rate of 0.0204 1/day in the process of biodegradable polymers composting. According to statistical analysis, the first-order kinetic model is suitable for the description of organic matter degradation (high R^2^ and EF and low RMSE values) during the grape skin composting process and can be used in the analysis of organic matter degradation dynamics.

### 3.7. Optimization of Composting Conditions

The aim of using response surface methodology was to determine optimal conditions for the grape skin composting process. As described by Asadu et al. [78], most often, response surface methodology is used to investigate the effects of independent variables on the response(s). It is also employed to consider the effects of several variables working together during composting process. In order to obtain substantial and highly stabilized compost, it is imperative to optimize operating conditions, as this is a critical step in process development and performance enhancement [79]. Composting is not a simple task because there are a lot of variables involved whereas RSM demonstrates a statistically sound approach for the fewest experiments possible [78]. In this work, the influence of initial moisture content (*X*_1_) and air flow rate (*X*_2_) on a compost organic matter amount at the end of the composting process (*Y*) was analyzed. A second-order polynomial was used to describe experimental data (Equation (9)) and the significant model coefficients (*p* < 0.05) are marked in bold.

(9) $$Y=73.749-8.445\cdot X_{1}-7.466\cdot X_{2}-17.411\cdot X_{1}^{2}-8.370\cdot X_{2}^{2}+0.54\cdot X_{1}\cdot X_{2}$$

**Figure 8:**
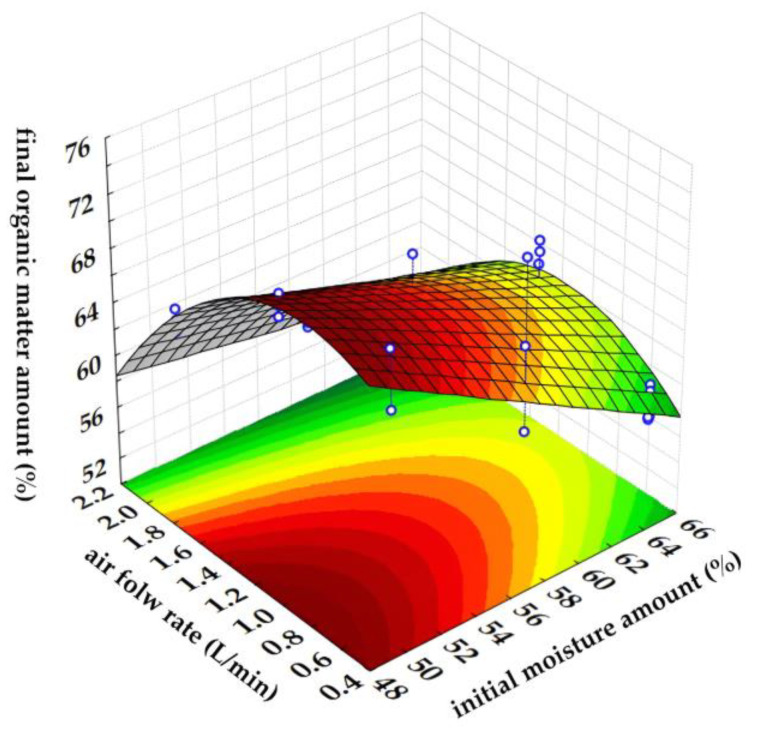
Response Surface Methodology (RSM) plot showing the interaction effects of initial moisture content and air flow rate on a final organic matter content.

The obtained results indicate that both variables, initial moisture content (X1), and air flow rate (X2) have a negative effect on the compost organic matter amount. This agrees with what is previously described, which is that the moisture content is over 65–70%, which can impair the movement of oxygen because the pore spaces are closed and the process can become anaerobic [27,31], and a high air flow rate can dry the composting mixture and reduce the microbial activity (Figure 8). Also, it can be noticed that initial moisture content and air flow rate interactions (X1·X2) have a positive effect on the compost organic matter amount. Statistical analysis of the model by the F test and the analysis of variance (Table 4) showed that the developed model is significant and can be used for the optimization of organic matter content. Furthermore, a lack of fit value (non-significant) showed that model coefficients are significant. The agreement between model experimental data and model predicted data was R^2^ = 0.8266.

Based on the desirability profile derived from the RSM predicted values, the composting conditions were optimized. The optimization matrix design revealed that the following conditions were necessary to obtain the minimum organic matter at the end of the composting process: 58.152% for the initial moisture content of the substrate and 1.0625 L/min for the air flow rate. A desirability scale ranging from 0 (undesirable, high organic matter content) to 1 (highly desirable, low organic matter content) was used. The proposed optimal experimental conditions predicted an organic matter content of 63.49% at the end of the composting procedure. The independent validation performed with optimal process conditions resulted in 60.157% of organic matter at the end of the composting process.

## 4. Conclusions

In the present work, nine composting experiments of grape skin were carried out under different conditions of initial moisture content and air flow rate in laboratory reactors. According to the results, grape skin waste can be transformed into compost through an environmentally friendly process. Our results showed the importance of initial moisture content and aeration rate on the process efficiency expressed as organic matter content. Furthermore, the obtained results showed that a first-order kinetics model can be used for the analysis of organic matter degradation dynamics. In future research, the development of the composting process of grape pomace in scale-up can significantly contribute to environmental protection and winery waste recycling.

## Figures and Tables

**Table 1:** Experimental conditions for grape skin composting process.

Experiment	Moisture Content (%)	Air Flow Rate (L/min)
1	50	0.50
2	50	1.25
3	65	0.88
4	65	1.40
5	65	0.35
6	50	2.00
7	57.5	1.70
8	57.5	0.43
9	57.5	1.06

**Table 2:** Bulk density and porosity of compost samples. Results are presented as average values ± standard deviation. (^a–d^ The same superscript lowercase letters denote no significant differences (*p* > 0.05) between values obtained for different composting processes according to Tukey’s ANOVA).

Experiment	Bulk Density ± S.D. (kg/m^3^)	Porosity ± S.D. (%)
1	323.466 ± 0.281 ^d^	73.388 ± 1.947 ^a^
2	323.803 ± 2.863 ^d^	73.563 ± 6.823 ^a^
3	388.119 ± 18.086 ^b^	63.777 ± 7.256 ^a^
4	393.146 ± 8.830 ^b^	64.862 ± 5.703 ^a^
5	396.708 ± 1.471 ^b^	63.509 ± 4.252 ^a^
6	384.481 ± 2.140 ^b^	66.613 ± 8.725 ^a^
7	368.571 ± 3.635 ^b,c^	65.668 ± 1.346 ^a^
8	358.508 ± 7.283 ^c^	68.209 ± 2.128 ^a^
9	428.805 ± 12.190 ^a^	61.257 ± 7.917 ^a^

**Table 3:** Kinetic parameters and statistical analysis for description of organic matter degradation.

	Exp.	*k* (1/day)	OM_0_ (%)	R^2^	R^2^_adj_	RMSE	c^2^	EF
Organic matter degradation	1	0.0035 ± 0.0014	77.2451 ± 1.8158	0.9148	0.8686	1.4083	1.4412	0.8908
	2	0.0032 ± 0.0013	79.7421 ± 1.8338	0.9281	0.8852	1.5169	1.8323	0.8834
	3	0.0093 ± 0.0023	80.4016 ± 2.9808	0.8798	0.8248	1.2285	1.8286	0.8668
	4	0.0056 ± 0.0016	77.1831 ± 2.0334	0.8752	0.8691	2.3197	2.6231	0.8042
	5	0.0052 ± 0.0013	75.3750 ± 1.7064	0.8256	0.8570	2.2491	2.2260	0.8364
	6	0.0037 ± 0.0010	72.2391 ± 1.2717	0.8826	0.8032	1.3768	1.3332	0.8439
	7	0.0073 ± 0.0010	72.5545 ± 1.2172	0.8887	0.8358	2.2731	2.3592	0.8887
	8	0.0014 ± 0.0013	70.9778 ± 1.5802	0.8680	0.8100	1.3753	2.3279	0.8688
	9	0.0034 ± 0.0008	71.5794 ± 0.9761	0.8976	0.8470	1.0316	1.3099	0.8919

**Table 4:** Analysis of variance for organic matter degradation optimization.

Source	SS	df	MS	F	p
*β*_1_	266.279	1	266.279	39.4026	0.00001
*β*_2_	42.870	1	42.870	6.3438	0.02146
*β*_11_	116.637	1	116.637	17.2593	0.00060
*β*_22_	44.350	1	44.350	6.5626	0.01961
*β*_12_	0.481	1	0.481	0.0711	0.04273
Lack-of-fit	173.625	3	57.875	8.5640	0.09611
Pure error	121.642	18	6.758		
Total SS	624.234	26			

## Data Availability

The original contributions presented in the study are included in the article/Supplementary Materials, further inquiries can be directed to the corresponding author.

## Supplementary Materials

The following supporting information can be downloaded at: https://www.mdpi.com/article/10.3390/foods13060824/s1, Table S1. Mean values of selected compost physicochemical properties during composting process with different initial moisture content and different aeration rate. Table S2. Mean values of germination indices during composting process.
