# Supplementary material for: Grape Skin Composting Process to Recycle Food Waste: Kinetics and Optimization
Source: Foods. 2024 Mar 7;13(6):824. doi: 10.3390/foods13060824 (PMC10968801; doi:10.3390/foods13060824)
Supplement: Supplementary file 1 [file foods-13-00824-s001.zip › foods-2893156-supplementary.pdf]

Table S1. Mean values of selected compost physicochemical properties during composting process with different initial moisture content and different aeration rate. $^{A-I}$ The same superscript capital letters within a row denote no significant differences (P > 0.05) between the values obtained for the different composting processes according to Tukey's ANOVA. $^{a-i}$. The same superscript lowercase letters within a column denote no significant differences (P > 0.05) between values obtained for different days of composting process according to Tukey's ANOVA.

		R1	R2	R3	R4	R5	R6	R7	R8	R9
Moisture	0	52.321±0.309^{A,a}	52.321±0.309^{A,a}	64.920±0.418^{B,a}	64.920±0.418^{B,a}	64.920±0.418^{B,a}	52.807±1.402^{A,a}	60.607±1.929^{B,a}	60.607±1.929^{B,a}	60.607±1.929^{B,a}
	4	51.615±0.666^{A,a}	52.337±3.482^{A,a}	61.375±2.073^{B,a}	61.585±5.162^{B,a}	64.153±0.844^{B,a}	50.991±1.236^{A,a}	58.864±1.292^{B,a}	64.462±0.813^{B,a}	63.167±0.529^{B,a}
	8	51.472±1.327^{A,a}	52.579±0.633^{A,a}	65.418±0.620^{B,a}	63.628±0.995^{B,a}	63.121±0.432^{B,a}	60.032±0.428^{B,b}	64.582±1.362^{B,a}	63.675±1.531^{B,a}	65.267±1.725^{B,a}
	12	54.133±0.093^{A,a}	52.931±3.006^{A,a}	66.411±0.254^{B,a}	67.414±0.818^{B,a}	65.518±2.846^{B,a}	57.157±0.771^{A,a}	67.687±1.358^{B,b}	60.771±0.768^{B,a}	65.160±0.987^{B,a}
	16	55.650±2.135^{A,a}	54.521±0.916^{A,a}	65.118±2.275^{B,a}	64.579±0.126^{B,a}	64.524±1.803^{B,a}	63.216±2.062^{B,b}	68.264±0.431^{B,b}	61.676±0.488^{B,a}	67.716±0.490^{B,b}
	20	55.753±1.272^{A,a}	53.602±0.994^{A,a}	68.570±1.812^{B,b}	66.747±3.639^{B,a}	66.310±0.261^{B,a}	60.021±0.660^{B,b}	65.385±1.729^{B,a}	64.625±0.488^{B,a}	66.088±2.200^{B,a}
	24	56.165±1.330^{A,a}	56.570±4.242^{A,a}	69.999±0.765^{B,b}	66.933±0.079^{B,a}	67.357±1.167^{B,a}	62.205±0.540^{B,b}	64.362±0.333^{B,a}	64.858±1.991^{B,a}	64.593±0.445^{B,a}
	30	56.457±0.412^{A,a}	55.580±0.237^{A,a}	70.776±0.028^{B,b}	68.412±0.717^{B,a}	69.332±0.096^{B,a}	62.922±0.208^{C,b}	70.671±0.507^{B,b}	65.630±2.874^{B,a}	67.763±1.646^{B,b}
Dry matter	0	47.679±0.309^{A,a}	47.679±0.309^{A,a}	35.080±0.419^{B,a}	35.080±0.419^{B,a}	35.080±0.419^{B,a}	47.197±1.402^{A,a}	39.393±1.929^{B,a}	39.393±1.929^{B,a}	39.393±1.929^{B,a}
	4	48.385±0.666^{A,a}	47.663±3.482^{A,a}	38.624±2.074^{B,a}	38.415±5.162^{B,a}	35.847±0.844^{B,a}	49.008±1.236^{A,a}	41.136±1.292^{B,a}	35.538±0.813^{B,a}	36.832±0.529^{B,a}
	8	48.528±1.327^{A,a}	47.421±0.623^{A,a}	34.582±0.620^{B,a}	36.372±0.995^{B,a}	36.878±0.433^{B,a}	39.967±0.428^{B,b}	35.417±1.362^{B,a}	36.325±1.531^{B,a}	34.733±1.752^{B,a}
	12	45.866±0.092^{A,a}	47.069±3.007^{A,a}	33.588±0.253^{B,a}	32.586±0.818^{B,a}	34.482±2.847^{B,a}	42.842±0.770^{A,a}	32.313±1.358^{B,b}	39.229±0.768^{C,a}	34.839±0.987^{B,a}
	16	44.349±2.135^{A,a}	45.479±0.916^{A,a}	34.882±2.275^{B,a}	35.421±0.125^{B,a}	35.476±1.804^{B,a}	36.784±2.062^{B,b}	31.736±0.431^{B,b}	38.324±0.488^{B,a}	32.284±0.490^{B,b}
	20	44.247±1.272^{A,a}	46.398±0.995^{A,a}	31.430±1.812^{B,a}	37.252±3.623^{B,a}	33.690±0.26^{B,a}	39.978±0.660^{A,b}	34.615±1.729^{B,a}	35.374±0.860^{B,a}	33.912±2.200^{B,a}
	24	46.435±2.964^{A,a}	43.429±4.242^{A,a}	35.001±0.759^{B,a}	35.066±0.079^{B,a}	32.643±1.167^{B,a}	37.795±0.540^{B,b}	35.638±0.333^{B,a}	38.142±1.991^{B,a}	35.407±0.446^{B,a}
	30	43.512±0.412^{A,a}	44.419±0.237^{A,a}	29.226±0.023^{B,a}	33.588±0.717^{B,a}	30.668±0.097^{B,a}	37.077±0.208^{B,b}	29.329±0.508^{B,b}	34.369±2.874^{B,a}	32.237±1.646^{B,b}
Organic matter	0	73.179±0.576^{A,a}	73.179±0.576^{A,a}	71.570±0.219^{A,a}	71.570±0.219^{A,a}	71.570±0.219^{A,a}	67.964±1.084^{A,a}	71.568±2.483^{A,a}	71.568±2.483^{A,a}	71.568±2.483^{A,a}
	4	73.078±3.521^{A,a}	74.283±1.815^{A,a}	70.051±4.409^{A,a}	72.587±3.097^{A,a}	71.765±1.527^{A,a}	73.931±1.527^{A,a}	74.285±0.267^{A,a}	68.568±1.013^{A,a}	69.351±0.075^{A,a}
	8	72.715±0.761^{A,a}	72.996±1.125^{A,a}	71.873±0.479^{A,a}	74.999±1.048^{A,a}	74.483±1.499^{A,a}	72.292±0.144^{A,a}	72.483±3.991^{A,a}	70.970±0.452^{A,a}	69.492±3.798^{A,a}
	12	76.312±3.395^{A,a}	73.075±3.671^{A,a}	65.521±2.688^{B,b}	63.849±0.661^{B,b}	66.812±4.574^{B,a}	70.605±1.061^{A,a}	61.679±0.414^{B,b}	70.839±1.141^{A,a}	68.534±1.203^{B,a}
	16	68.742±2.194^{A,a}	72.719±0.379^{A,a}	64.394±0.336^{B,b}	69.842±1.877^{A,a}	69.087±4.155^{A,a}	64.802±0.576^{B,a}	62.104±0.088^{B,b}	67.743±2.877^{A,a}	65.337±0.753^{B,b}
	20	71.046±0.503^{A,a}	71.405±1.032^{A,a}	63.971±1.484^{B,b}	69.421±5.201^{A,a}	69.766±0.537^{A,a}	68.527±1.081^{A,a}	60.954±0.954^{B,b}	67.491±3.128^{A,a}	66.479±2.067^{A,a}
	24	69.409±1.444^{A,a}	70.807±4.897^{A,a}	64.908±0.193^{B,b}	68.638±0.703^{A,a}	68.392±3.522^{A,a}	67.212±3.172^{A,a}	63.202±2.119^{B,b}	66.922±2.777^{A,a}	66.664±1.338^{A,a}
	30	68.785±2.176^{A,a}	69.273±0.824^{A,a}	57.319±0.129^{B,c}	64.576±0.886^{B,b}	59.316±0.509^{B,b}	62.924±0.576^{A,a}	58.263±0.293^{B,b}	67.221±6.309^{A,a}	64.944±3.706^{A,b}

**Ash**	0	26.821±0.576^{A,a}	26.821±0.576^{A,a}	28.429±0.219^{A,b}	28.429±0.219^{A,a}	28.429±0.219^{A,a}	32.035±1.084^{A,a}	28.431±2.482^{A,a}	28.431±2.482^{A,a}	28.431±2.482^{A,a}
	4	26.921±3.521^{A,a}	25.716±1.816^{A,a}	22.949±4.409^{A,b}	19.912±5.598^{A,b}	28.234±1.527^{A,a}	26.068±1.527^{A,a}	25.714±0.267^{A,a}	31.432±1.013^{A,a}	30.649±0.075^{A,a}
	8	17.285±0.760^{A,b}	17.004±1.125^{A,b}	15.122±0.479^{A,c}	25.001±1.048^{A,a}	25.516±1.499^{A,a}	27.708±0.144^{B,a}	27.517±3.990^{B,a}	29.029±0.453^{B,a}	30.506±3.798^{B,a}
	12	23.687±3.396^{A,a}	24.924±3.672^{A,a}	28.429±0.219^{B,b}	36.153±0.661^{B,a}	33.187±4.574^{B,a}	29.349±1.061^{A,a}	38.320±0.414^{B,b}	29.160±1.404^{A,a}	25.465±1.203^{A,a}
	16	31.258±2.194^{A,a}	26.280±0.379^{A,a}	34.478±2.688^{A,b}	29.735±0.475^{A,a}	26.912±4.156^{A,a}	35.197±0.576^{A,a}	37.895±0.089^{A,b}	22.667±0.189^{B,a}	34.662±0.753^{A,a}
	20	28.954±0.503^{A,a}	22.595±1.033^{A,a}	35.605±0.337^{A,b}	30.579±5.201^{A,a}	30.264±0.537^{A,a}	31.473±1.081^{A,a}	39.045±0.955^{B,b}	32.509±3.129^{A,a}	33.521±2.066^{A,a}
	24	24.069±3.440^{A,a}	26.193±4.897^{A,a}	35.092±0.193^{B,b}	31.362±0.703^{A,a}	31.608±3.522^{A,a}	32.787±3.172^{B,a}	36.797±0.080^{B,a}	33.078±2.777^{B,a}	33.335±1.338^{B,a}
	30	30.682±2.176^{A,a}	30.726±0.824^{A,a}	42.688±0.129^{B,a}	35.423±0.886^{A,a}	40.684±0.509^{B,a}	37.078±0.848^{A,a}	41.736±0.293^{B,b}	32.779±6.309^{A,a}	35.056±3.706^{A,a}
**Carbon**	0	48.8±0.4^{B,b}	48.8±0.4^{B,b}	49.6±0.4^{A,b}	49.6±0.4^{A,b}	49.6±0.4^{A,b}	47.7±0.4^{B,b}	48.1±0.4^{B,a}	48.1±0.4^{B,a}	48.1±0.4^{B,a}
	4	50.8±0.4^{A,a}	51.8±0.4^{A,a}	52.2±0.4^{A,a}	50.8±0.4^{B,a}	50.9±0.4^{A,a}	48.1±0.4^{B,a}	47.9±0.4^{B,a}	48.5±0.4^{B,a}	47.6±0.4^{B,a}
	8	51.4±0.4^{A,a}	51.2±0.4^{A,a}	51.5±0.4^{A,a}	51.2±0.4^{A,a}	51.3±0.4^{A,a}	47.9±0.4^{B,a}	47.3±0.4^{B,a}	48.1±0.4^{B,a}	48.0±0.4^{B,a}
	12	49.0±0.4^{A,b}	49.9±0.4^{A,a}	48.1±0.4^{A,c}	51.4±0.4^{A,a}	49.3±0.4^{A,a}	44.3±0.4^{B,c}	46.8±0.4^{B,a}	47.4±0.4^{B,a}	46.3±0.4^{B,a}
	16	48.7±0.4^{A,b}	48.4±0.4^{A,b}	49.3±0.4^{A,b}	49.4±0.4^{A,b}	48.3±0.4^{A,b}	46.6±0.4^{B,b}	47.1±0.4^{B,a}	47.7±0.4^{B,a}	47.2±0.4^{B,a}
	20	48.7±0.4^{A,b}	48.4±0.4^{A,b}	47.1±0.4^{B,c}	47.4±0.4^{A,b}	47.2±0.4^{B,c}	48.1±0.4^{A,a}	46.3±0.4^{B,a}	48.2±0.4^{A,a}	48.0±0.4^{A,a}
	24	48.3±0.4^{A,b}	48.7±0.4^{A,b}	47.5±0.4^{A,c}	47.3±0.4^{A,b}	47.5±0.4^{A,c}	47.2±0.4^{A,b}	47.0±0.4^{A,a}	47.2±0.4^{A,a}	47.3±0.4^{A,a}
	30	48.3±0.4^{A,b}	47.7±0.4^{A,b}	46.8±0.4^{B,c}	47.3±0.4^{A,b}	47.3±0.4^{A,c}	47.1±0.4^{A,b}	44.7±0.4^{A,b}	47.3±0.4^{A,a}	47.3±0.4^{A,a}
**Nitrogen**	0	1.78±0.4^{A,a}	1.78±0.4^{A,a}	1.27±0.4^{A,a}	1.27±0.4^{A,a}	1.27±0.4^{A,a}	1.54±0.4^{A,a}	1.50±0.4^{A,a}	1.50±0.4^{A,a}	1.50±0.4^{A,a}
	4	1.86±0.4^{A,a}	1.85±0.4^{A,a}	1.84±0.4^{A,a}	1.73±0.4^{A,a}	1.83±0.4^{A,a}	1.48±0.4^{A,a}	1.57±0.4^{A,a}	1.58±0.4^{A,a}	1.61±0.4^{A,a}
	8	1.86±0.4^{A,a}	1.93±0.4^{A,a}	2.06±0.4^{A,a}	1.90±0.4^{A,a}	1.84±0.4^{A,a}	1.55±0.4^{A,a}	1.43±0.4^{A,a}	1.61±0.4^{A,a}	1.60±0.4^{A,a}
	12	1.83±0.4^{A,a}	1.83±0.4^{A,a}	1.96±0.4^{A,a}	1.77±0.4^{A,a}	1.91±0.4^{A,a}	1.96±0.4^{A,a}	2.08±0.4^{A,a}	1.71±0.4^{A,a}	2.56±0.4^{A,a}
	16	1.82±0.4^{A,a}	1.87±0.4^{A,a}	1.85±0.4^{A,a}	2.01±0.4^{A,a}	1.99±0.4^{A,a}	2.76±0.4^{A,a}	2.31±0.4^{A,a}	1.93±0.4^{A,a}	2.67±0.4^{A,a}
	20	1.66±0.4^{A,a}	1.72±0.4^{A,a}	1.92±0.4^{A,a}	1.88±0.4^{A,a}	1.77±0.4^{A,a}	2.17±0.4^{A,a}	2.53±0.4^{A,a}	1.80±0.4^{A,a}	2.20±0.4^{A,a}
	24	1.66±0.4^{A,a}	1.82±0.4^{A,a}	1.90±0.4^{A,a}	1.75±0.4^{A,a}	1.77±0.4^{A,a}	2.36±0.4^{A,a}	2.56±0.4^{A,a}	1.87±0.4^{A,a}	2.19±0.4^{A,a}
	30	1.57±0.4^{A,a}	1.67±0.4^{A,a}	1.90±0.4^{A,a}	1.87±0.4^{A,a}	1.76±0.4^{A,a}	2.33±0.4^{A,a}	2.84±0.4^{A,a}	2.07±0.4^{A,a}	2.51±0.4^{A,a}
**C/N ratio**	0	27.947±6.210^{A,a}	27.947±6.210^{A,a}	40.074±7.347^{B,a}	40.074±7.347^{B,a}	40.074±7.347^{B,a}	32.419±8.442^{A,a}	33.652±9.023^{A,a}	33.652±9.023^{A,a}	33.652±9.023^{A,a}
	4	28.164±5.978^{A,a}	28.883±6.172^{A,a}	29.276±6.294^{A,b}	30.672±7.047^{A,b}	28.711±6.204^{A,b}	34.154±9.295^{A,a}	31.875±8.126^{A,a}	32.052±8.118^{A,a}	30.818±7.641^{A,a}
	8	28.495±6.052^{A,a}	27.292±5.568^{A,a}	25.629±4.873^{A,b}	27.858±5.781^{A,b}	28.769±6.204^{A,b}	32.325±8.358^{A,a}	34.892±9.860^{A,a}	31.142±7.724^{A,a}	31.289±7.813^{A,a}
	12	27.638±5.963^{A,a}	27.505±5.801^{A,a}	25.223±5.047^{A,b}	28.875±6.462^{A,b}	26.570±5.474^{A,b}	23.228±4.632^{A,b}	23.050±4.319^{A,b}	28.750±6.672^{A,a}	18.371±2.747^{B,b}
	16	28.628±5.996^{A,a}	29.603±8.521^{A,a}	27.487±5.862^{A,b}	25.225±4.917^{A,b}	24.925±4.907^{A,b}	17.111±2.359^{B,b}	20.789±3.478^{A,b}	25.424±5.173^{A,a}	17.933±2.565^{B,b}
	20	30.502±7.319^{A,a}	27.459±5.957^{A,a}	25.243±5.162^{A,b}	25.978±5.436^{A,b}	27.588±6.164^{A,b}	22.662±4.066^{A,b}	18.596±2.817^{B,b}	27.671±6.075^{A,a}	22.293±3.936^{A,b}
	24	26.775±5.673^{A,a}	28.814±6.526^{A,a}	25.742±5.325^{A,b}	26.169±6.609^{A,b}	27.763±6.205^{A,b}	20.374±3.331^{A,b}	18.649±2.792^{B,b}	26.015±5.475^{A,a}	22.399±3.974^{A,b}
	30	26.142±8.169^{A,a}	27.125±6.156^{A,a}	24.673±4.959^{A,b}	27.174±5.729^{A,b}	28.168±6.336^{A,b}	21.041±3.491^{A,b}	17.365±2.328^{B,b}	23.960±4.521^{A,b}	19.438±2.975^{A,b}

ΔE (compost)	2	2.436±0.332^{A,a}	2.246±2.083^{A,a}	3.552±0.359^{A,a}	2.912±1.259^{A,a}	3.353±0.035^{A,a}	2.930±0.435^{A,a}	2.629±1.487^{A,a}	4.139±1.922^{A,a}	5.910±1.951^{A,a}
	8	9.489±2.572^{A,b}	11.485±2.912^{A,b}	11.248±1.567^{A,b}	8.153±0.154^{A,b}	13.024±3.321^{A,b}	7.097±0.006^{A,b}	7.080±1.251^{A,b}	10.832±0.507^{A,b}	6.719±0.791^{A,a}
	12	11.169±3.836^{A,b}	16.856±0.707^{B,c}	16.459±0.392^{B,c}	10.565±0.051^{A,b}	13.206±0.011^{A,b}	9.038±0.303^{A,b}	9.653±0.701^{A,b}	9.495±0.833^{A,b}	8.109±0.155^{A,a}
	16	14.392±2.048^{A,b}	12.912±2.212^{A,b}	15.740±0.553^{A,c}	11.455±0.060^{A,b}	13.425±0.661^{A,b}	7.302±0.219^{B,b}	9.141±1.130^{B,b}	8.460±1.077^{B,a}	7.713±1.038^{B,a}
	20	14.216±2.225^{A,b}	14.435±2.361^{A,b}	15.348±0.354^{A,b}	12.952±0.195^{A,b}	13.719±0.074^{A,b}	6.943±0.431^{B,b}	8.520±1.466^{B,b}	7.184±1.245^{B,a}	7.826±0.873^{B,a}
	24	15.201±2.267^{A,c}	10.392±3.529^{A,b}	14.855±0.314^{A,b}	13.198±0.150^{A,c}	13.542±0.029^{A,b}	7.366±0.096^{B,b}	7.876±1.596^{B,b}	7.929±1.394^{B,a}	7.802±1.323^{B,a}
	30	16.003±1.057^{A,c}	9.970±2.078^{B,b}	15.302±0.269^{B,b}	13.411±0.10^{B,c}	14.387±0.327^{B,b}	6.593±0.822^{B,b}	7.721±1.487^{B,b}	7.841±1.147^{B,a}	8.377±1.045^{B,a}
pH	0	7.683±0.005^{A,a}	7.683±0.005^{A,a}	7.647±0.006^{A,a}	7.647±0.006^{A,a}	7.647±0.006^{A,a}	5.803±0.006^{B,a}	5.623±0.006^{B,a}	5.623±0.006^{B,a}	5.623±0.006^{B,a}
	4	5.297±0.002^{A,b}	4.877±0.021^{B,b}	5.140±0.010^{C,b}	4.990±0.030^{D,b}	4.767±0.006^{E,b}	5.710±0.010^{F,b}	5.510±0.010^{G,b}	7.450±0.010^{H,b}	5.707±0.006^{I,b}
	8	4.773±0.001^{A,c}	5.043±0.006^{B,c}	6.550±0.001^{C,c}	5.463±0.006^{D,c}	6.157±0.006^{E,c}	7.840±0.036^{F,c}	7.103±0.005^{G,c}	7.533±0.015^{H,c}	6.907±0.006^{I,c}
	12	6.297±0.001^{A,d}	7.367±0.006^{B,d}	7.080±0.017^{C,c}	6.103±0.006^{D,d}	7.307±0.006^{E,d}	7.080±0.017^{F,d}	8.753±0.015^{G,d}	7.213±0.005^{H,d}	7.480±0.020I^{,d}
	16	7.633±0.012^{A,a}	7.163±0.011^{B,d}	8.333±0.006^{C,d}	7.383±0.006^{D,e}	8.067±0.006^{E,e}	7.483±0.015^{F,e}	8.790±0.020^{G,d}	7.573±0.032^{H,c}	8.287±0.015^{I,e}
	20	8.070±0.006^{A,e}	7.750±0.021^{B,a}	9.240±0.010^{C,e}	7.567±0.006^{D,e}	8.200±0.010^{E,f}	8.393±0.006^{F,f}	8.913±0.012^{G,e}	7.523±0.006^{H,c}	8.390±0.010^{I,f}
	24	7.357±0.015^{A,f}	7.257±0.005^{B,d}	8.563±0.010^{C,e}	8.210±0.010^{D,f}	8.260±0.010^{E,f}	8.420±0.026^{F,g}	8.800±0.015^{G,f}	7.787±0.006^{H,e}	8.600±0.023^{I,g}
	30	7.297±0.006^{A,f}	7.303±0.006^{B,d}	9.080±0.010^{C,e}	7.833±0.015^{D,g}	7.943±0.015^{E,g}	8.483±0.035^{F,h}	9.243±0.021^{G,g}	8.003±0.012^{H,f}	8.587±0.006^{I,g}
TDS	0	812±15^{A,b}	812±15^{A,c}	484±2^{B,d}	484±2^{B,a}	484±2^{B,a}	1390±40^{C,a}	1178±20^{C,a}	1177±19^{C,a}	1177±19^{C,a}
	4	655±5^{A,b}	386±2^{B,c}	799±21^{C,c}	574±7^{A,a}	481±2^{B,a}	1030±18^{D,b}	1836±2^{C,b}	1261±10^{D,a}	810±9^{C,b}
	8	543±47^{A,c}	445±5^{A,c}	432±6^{A,d}	574±2^{A,a}	519±3^{A,a}	1007±13^{B,b}	944±16^{C,b}	1594±4^{D,d}	1212±10^{E,a}
	12	643±14^{A,a}	1476±4^{B,a}	1219±3^{A,b}	1481±1^{B,b}	1004±1^{C,b}	1062±16^{A,b}	1843±29^{E,c}	1116±14^{A,a}	1771±17^{D,c}
	16	1540±13^{A,a}	1228±27^{A,b}	1062±1^{B,b}	1036±4^{B,c}	1331±3^{B,a}	1088±60^{B,b}	970±5^{B,d}	1054±2^{B,a}	1500±43^{A,c}
	20	1535±9^{A,a}	1292±7^{B,b}	891±5^{C,c}	714±24^{C,d}	784±24^{D,c}	1880±40^{E,c}	1172±15^{D,b}	966±3^{C,b}	1672±14^{A,c}
	24	1455±12^{A,a}	1639±4^{B,a}	1218±4^{A,b}	1449±18^{A,b}	1387±19^{A,b}	2227±11^{C,d}	1387±40^{A,a}	1465±7^{A,d}	1830±14^{B,c}
	30	1303±25^{A,a}	1466±3^{A,b}	1409±18^{A,a}	1458±5^{A,b}	1134±10^{A,b}	1965±27^{B,c}	1574±24^{A,a}	1204±36^{A,c}	2097±16^{B,c}
S	0	1666±6^{A,b}	1666±6^{A,c}	966±15^{B,d}	966±15^{B,a}	966±15^{B,a}	2780±80^{C,a}	2413±6^{C,a}	2413±6^{C,a}	2413±6^{C,a}
	4	1310±11^{A,b}	781±17^{B,c}	1597±41^{C,c}	1149±13^{A,a}	964±3^{B,a}	2060±36^{D,b}	1667±2^{C,b}	2367±61^{D,a}	1576±10^{C,b}
	8	1092±82^{A,c}	890±10^{A,c}	871±17^{A,d}	1096±4^{A,a}	1048±5^{A,a}	2014±25^{B,b}	1957±10^{C,b}	3173±6^{D,d}	2447±6^{E,a}
	12	2295±18^{A,a}	2957±6^{B,a}	2443±15^{A,b}	2983±15^{B,b}	2017±6^{C,b}	2123±31^{A,b}	3907±32^{E,c}	2170±30^{A,a}	3243±92^{D,c}
	16	2780±12^{A,a}	2507±6^{A,b}	2077±15^{B,b}	2103±6^{B,c}	2587±35^{A,b}	2177±119^{B,b}	1912±17^{B,b}	1933±19^{B,a}	3000±82^{A,c}
	20	3120±11^{A,a}	2493±40^{B,b}	1737±31^{C,c}	1481±1^{C,d}	1669±11^{D,c}	3670±79^{E,c}	2193±132^{D,b}	1865±31^{C,b}	3273±40^{A,c}
	24	2877±112^{A,a}	3283±6^{B,a}	2270±101^{A,b}	2987±6^{A,b}	2680±46^{A,b}	4453±21^{C,d}	2640±40^{A,a}	2970±10^{A,d}	3513±61^{B,c}
	30	2663±6^{A,a}	2757±222^{A,b}	2860±10^{A,a}	2783±136^{A,b}	2320±10^{A,b}	3930±53^{B,c}	2877±71^{A,a}	2530±10^{A,c}	4013±96^{B,d}

ΔE (extract)	2	$0.691±0.029^{A,a}$	$0.807±0.023^{A,a}$	$0.338±0.008^{A,a}$	$0.206±0.011^{A,a}$	$0.367±0.008^{A,a}$	$2.840±0.035^{B,a}$	$1.061±0.122^{A,a}$	$0.338±0.133^{A,a}$	$0.795±0.067^{A,a}$
	8	$1.073±0.335^{A,a}$	$0.406±0.276^{A,a}$	$0.280±0.005^{A,a}$	$0.346±0.005^{A,a}$	$0.374±0.002^{A,a}$	$2.110±0.166^{B,a}$	$4.093±0.181^{C,b}$	$3.007±0.123^{D,b}$	$2.843±0.237^{B,b}$
	12	$1.535±.0027^{A,b}$	$1.429±0.498^{A,a}$	$1.342±0.042^{A,b}$	$1.080±0.002^{A,b}$	$1.757±0.010^{A,b}$	$1.775±0.032^{A,b}$	$1.447±0.077^{A,a}$	$1.315±0.082^{A,c}$	$0.983±0.048^{A,a}$
	16	$0.789±0.057^{A,a}$	$1.210±0.459^{A,a}$	$1.518±0.011^{A,b}$	$1.370±0.014^{A,b}$	$1.087±0.010^{A,b}$	$5.244±0.058^{B,d}$	$4.135±0.155^{C,b}$	$2.292±0.107^{D,b}$	$2.887±0.078^{D,b}$
	20	$0.818±0.026^{A,a}$	$0.981±0.041^{A,a}$	$1.419±0.015^{A,b}$	$1.560±0.006^{A,b}$	$1.500±0.028^{A,b}$	$2.782±0.043^{B,a}$	$0.204±0.060^{A,c}$	$0.254±0.010^{A,a}$	$2.387±0.157^{B,b}$
	24	$1.487±0.003^{A,b}$	$2.040±0.411^{A,b}$	$1.813±0.026^{A,b}$	$1.913±0.006^{A,c}$	$2.022±0.065^{A,c}$	$0.572±0.021^{B,c}$	$1.642±0.117^{A,a}$	$3.283±0.096^{C,b}$	$2.148±0.132^{A,c}$
	30	$3.427±1.370^{A,c}$	$2.013±0.332^{B,b}$	$3.679±0.021^{A,c}$	$1.954±0.016^{B,c}$	$2.437±0.132^{B,c}$	$1.500±0.046^{B,b}$	$3.739±0.120^{A,b}$	$3.895±0.110^{A,d}$	$3.334±1.286^{A,d}$

Table S2. Mean values of germination indices during composting process. $^{A-I}$ The same superscript capital letters within a row denote no significant differences ($P > 0.05$) between the values obtained for the different composting processes according to Tukey's ANOVA. $^{a-i}$. The same superscript lowercase letters within a column denote no significant differences ($P > 0.05$) between values obtained for different days of composting process according to Tukey's ANOVA.

	R1	R2	R3	R4	R5	R6	R7	R8	R9
0	$56.135±3.850^{A,c}$	$56.135±2.370^{A,b}$	$0.000±0^{B,d}$	$0.000±0^{B,d}$	$0.000±0^{B,c}$	$0.000±0^{B,e}$	$0.000±0^{B,d}$	$0.000±0^{B,d}$	$0.000±0^{B,d}$
6	$88.978±2.629^{B,b}$	$81.458±7.892^{B,a}$	$100.031±2.044^{B,a}$	$106.904±2.431^{B,b}$	$93.198±3.713^{B,b}$	$69.846±2.732^{C,c}$	$80.704±4.993^{B,c}$	$100.650±9.778^{B,c}$	$125.742±6.485^{A,b}$
10	$96.182±13.013^{B,b}$	$88.478±5.610^{B,a}$	$79.249±3.584^{B,b}$	$79.249±2.431^{B,c}$	$122.260±3.572^{A,a}$	$61.461±8.298^{C,c}$	$71.271±4.545^{B,c}$	$139.398±7.228^{A,b}$	$76.014±3.994^{B,c}$
16	$98.507±5.880^{C,b}$	$73.853±7.270^{D,a}$	$44.581±3.497^{E,c}$	$89.240±2.251^{C,c}$	$83.944±3.094^{C,b}$	$154.040±7.276^{A,a}$	$167.641±8.937^{A,a}$	$163.473±5.637^{A,a}$	$127.665±7.078^{B,b}$
22	$116.980±7.294^{B,a}$	$87.015±3.908^{C,a}$	$94.674±5.473^{B,a}$	$68.477±1.953^{D,c}$	$90.817±3.832^{C,b}$	$110.051±6.409^{B,b}$	$134.158±7.723^{A,b}$	$131.307±6.897^{A,b}$	$65.811±6.841^{D,c}$
26	$56.958±3.038^{D,c}$	$96.960±9.741^{B,a}$	$92.280±3.875^{B,a}$	$109.562±2.411^{B,b}$	$80.049±4.196^{C,b}$	$36.684±3.277^{E,d}$	$124.695±5.606^{A,b}$	$108.270±4.136^{B,c}$	$80.704±7.142^{C,c}$
30	$96.921±9.547^{D,b}$	$81.627±3.277^{D,a}$	$79.295±3.277^{D,b}$	$154.711±1.818^{B,a}$	$133.859±4.993^{C,a}$	$74.211±5.730^{E,c}$	$147.509±7.392^{C,b}$	$147.720±2.875^{C,a}$	$202.426±10.565^{A,a}$
